# Reconciling model predictions with low reported cases of COVID-19 in Sub-Saharan Africa: insights from Madagascar

**DOI:** 10.1080/16549716.2020.1816044

**Published:** 2020-10-05

**Authors:** Michelle V. Evans, Andres Garchitorena, Rado J. L. Rakotonanahary, John M. Drake, Benjamin Andriamihaja, Elinambinina Rajaonarifara, Calistus N. Ngonghala, Benjamin Roche, Matthew H. Bonds, Julio Rakotonirina

**Affiliations:** a Odum School of Ecology and Center for the Ecology of Infectious Diseases, University of Georgia, Athens, GA, USA; b MIVEGEC, Ecole Pierre Louis de Santé Publique, Université de Montpellier, CNRS, IRD, Montpellier, France; c PIVOT, Ranomafana, Madagascar; d Madagascar Institut pour la Conservation des Ecosystèmes Tropicaux, Antananarivo, Madagascar; e Sorbonne Universite, Paris, France; f Department of Mathematics and Emerging Pathogens Institute, University of Florida, Gainesville, FL, USA; g IRD, Sorbonne Université, UMMISCO, Bondy, France; h Universidad Nacional Autónoma de México, Ciudad de México, México; i Harvard Medical School, Boston, MA, USA; j Faculty of Medicine, University of Antananarivo, Antananarivo, Madagascar

**Keywords:** COVID-19, Madagascar, infectious disease modelling, non-pharmaceutical interventions, age-structured contacts, outbreak response

## Abstract

COVID-19 has wreaked havoc globally with particular concerns for sub-Saharan Africa (SSA), where models suggest that the majority of the population will become infected. Conventional wisdom suggests that the continent will bear a higher burden of COVID-19 for the same reasons it suffers from other infectious diseases: ecology, socio-economic conditions, lack of water and sanitation infrastructure, and weak health systems. However, so far SSA has reported lower incidence and fatalities compared to the predictions of standard models and the experience of other regions of the world. There are three leading explanations, each with different implications for the final epidemic burden: (1) low case detection, (2) differences in epidemiology (e.g. low *R _0_*), and (3) policy interventions. The low number of cases have led some SSA governments to relaxing these policy interventions. Will this result in a resurgence of cases? To understand how to interpret the lower-than-expected COVID-19 case data in Madagascar, we use a simple age-structured model to explore each of these explanations and predict the epidemic impact associated with them. We show that the incidence of COVID-19 cases as of July 2020 can be explained by any combination of the late introduction of first imported cases, early implementation of non-pharmaceutical interventions (NPIs), and low case detection rates. We then re-evaluate these findings in the context of the COVID-19 epidemic in Madagascar through August 2020. This analysis reinforces that Madagascar, along with other countries in SSA, remains at risk of a growing health crisis. If NPIs remain enforced, up to 50,000 lives may be saved. Even with NPIs, without vaccines and new therapies, COVID-19 could infect up to 30% of the population, making it the largest public health threat in Madagascar for the coming year, hence the importance of clinical trials and continually improving access to healthcare.

## Background

The COVID-19 pandemic has killed hundreds of thousands of people, collapsing health systems and economies around the world. Most models predict that without intervention, the majority of the global population will become infected and tens of millions will die as a result of the pandemic [1]. There have been particular concerns for sub-Saharan Africa (SSA) [2–4], as the major factors that drive high burdens of other infectious diseases, such as the environmental and socio-economic conditions, lack of water and sanitation infrastructure, and weak health systems, are equally relevant to the threat of COVID-19. However, so far, the perceived burden of COVID-19 in SSA is low compared to expectations both from epidemiological models and from epidemic patterns in other regions of the world [5,6]. Though SSA comprises 11% of the global population, it comprised only 3.6% of the total global COVID-19 incidence during the first 5 months of the pandemic, much of which was due to case reports from South Africa [7]. As of July 2020, most SSA countries are reporting fewer than 100 new cases daily [8]. There are three leading potential explanations for the lower observed burden of COVID-19 in SSA: 1) low case detection, 2) region-specific epidemiology (e.g. different *R*_0_), and 3) early implementation of effective policy interventions. The important difference among these alternative explanations is that explanations based on low case detection and effective interventions imply that there will be a major resurgence if interventions are relaxed, while explanations based on region-specific epidemiology allow for a safe reopening.

The lower-than-expected number of reported cases may be due to low detection and reporting rates. RT-PCR laboratory capacity in SSA is limited [9] and many countries have among the lowest testing rates in the world [8]. Moreover, health-care access for fever and respiratory infections is low [10], which means that many symptomatic cases will not be detected, and the stigma associated with COVID-19 could further reduce health-seeking behaviors [11].

The epidemiology-based explanations for low COVID-19 cases are based on considerations of well-established factors: warmer climates, younger age distributions, and lower contact rates due to lower population density and transportation infrastructure in rural areas [12,13]. In addition, there is considerable interest in the potential immune-mediated consequences from living in a system with greater exposure to other infectious diseases and related prophylaxis and therapeutics [14]. For example, there are major trials underway on the effects of trained immunity due to the BCG vaccine, which may increase innate immunity against a range of respiratory infections [15]. However, many of these hypotheses have recently come into doubt. The pandemic phase of COVID-19 is driven by high susceptibility, not climate [16], suggesting that warmer, humid climates will not decrease transmission at this time. Further, past outbreaks of influenza, including the 1968 pandemic and 2009 H1N1 outbreak, spread throughout the African continent and were not limited by sparse transportation networks [17]. Explanations based in region-specific epidemiology are therefore only weakly supported.

The policy response in Africa has also been a source of considerable optimism [5,18]. African governments implemented early and strong non-pharmaceutical intervention (NPI) policies that may have effectively contained disease transmission [5,19]. The first case of COVID-19 was reported in SSA one month later than the first cases in Europe, allowing countries to prepare and implement NPIs, particularly lockdowns, social distancing, masks, and regulated domestic travel, during the early stages of the pandemic [3,19,20]. Beginning in June, several SSA countries began relaxing lockdown NPIs in response to the economic and social costs of lockdown given the low reported case numbers. As partial lockdowns have been lifted, some countries’ case rates have remained stable, while others have begun to increase, leading to the WHO to urge caution and emphasize the need for a gradual and conservative release of confinement measures in SSA [21].

It remains unknown whether SSA-specific conditions will result in different epidemic dynamics in SSA than elsewhere, and whether the current lower-than-expected case burdens can be explained solely by detection rates and policies. To explore these issues, we compare COVID-19 reported case data with predictions from a simple Susceptible-Exposed-Infectious-Recovered compartmental model for Madagascar that integrates age-structured social contact matrices and fatality rates, assuming a basic reproduction number, *R_0_*, of 2.5 [22–24] (Appendix A). We then consider possible levels of detection or NPI effectiveness that could explain the current state of the epidemic, and whether those levels are plausible given Madagascar’s policies, demographics, and environmental context. Finally, based on these explanations and the model framework, we investigate possible transmission scenarios for the first year of the epidemic. In particular, we examine the future of COVID-19 dynamics and control in Madagascar, which could be applicable to other SSA countries. Importantly, this simple model serves as a tool to further support the qualitative evidence outlined below, rather than as a predictive technique.

Madagascar’s demographic, economic, and health system profile is comparable to many other SSA countries [23,25,26]. It shares most of the major infectious diseases of mainland Africa (e.g. tuberculosis, malaria, respiratory infections, diarrheal diseases), and has recently endured among the worst epidemics of plague and measles in decades [27,28]. Like other SSA countries, Madagascar reported its first imported case relatively late, on 20 March 2020, and the government implemented NPIs early in the epidemic (Table 1). Madagascar instituted a national lockdown on 23 March 2020, three days before its first case attributed to local transmission. Testing practices are also similar to those in other SSA countries, initially focusing on screening for imported cases and eventually expanding to test contacts of known cases for local transmission.

**Table 1.** Timeline of non-pharmaceutical interventions (NPIs) implemented in Madagascar.

Date	Policy/Intervention/Program	Geographic extent (Region)
By March 19	Allowing the remaining people outside of the country and willing to return to Madagascar to come back	National level
March 20	Beginning of the epidemic in Madagascar with 3 initial imported cases announced	Analamanga*
March 20	Interruption of all international and regional flights from the outside of the country	National level
March 21	Following-up and testing all passengers entering Madagascar for the last 14 days for COVID-19	National level
March 23	Lockdown; curfew; interruption of all public transportation (ground and air travel) connecting Antananarivo and Toamasina to the other regions with establishment of health barrier at all national roads; prohibition of all meeting for more that 50 individuals	Analamanga and Atsinanana**
March 26	Reception of 20,000 Antibody RDT kits for COVID-19 testing	NA
March 31 to April 02	Mass Antibody RDT testing for all passengers entering Madagascar on March 11–19	Analamanga and Atsinanana
April 03	Adding the lockdown to Fianarantsoa after detection of COVID-19 confirmed cases	Haute-Matsiatra***, Analamanga and Atsinanana
April 05	Continuing lockdown and curfew	Analamanga, Atsinanana and Haute-Matsiatra
April 07–09	Temporary opening of national transportation by taxi-brousse to allow people from the other regions but blocked in other cities to return home	National level
April 17	Partial lifting of lockdown, which allow people to go out, as well as taxi and bus to work from 6:00 a.m. to 1:00 p.m; curfew maintained; social distancing and mandatory wearing of mask for all person going out	Analamanga, Atsinanana and Haute-Matsiatra
April 20	Launching the COVID Organics tisane based on Artemisia	NA
April 22	All classes preparing official exam resume	National level
May 04	All previous measures maintained, and adding the Alaotra-Mangoro region; church could receive no more than 50 persons	Analamanga, Atsinanana, Haute-Matsiatra and Alaotra-Mangoro****
May 04	Church could receive no more than 200 persons	The remaining 18 regions
May 17	All previous measures maintained	Analamanga, Atsinanana, Haute-Matsiatra and Alaotra-Mangoro
May 31	Progressive lifting of lockdown allowing people to work from 6:00 a.m to 3:00 p.m	Analamanga
May 31	Toamasina region totally locked to the other location, all classes interrupted, and people can work from 6:00 a.m. to 1:00 p.m	Atsinanana
May 31	All previous measures maintained	Alaotra-Mangoro
May 31	Daily life return to the normal because COVID-19 is controlled in Fianarantsoa	Haute-Matsiatra
June 14	Progressive lifting of lockdown allowing people to work from 6:00 a.m to 5:00 p.m, and public transportation until 7:00 p.m	Analamanga
June 14	Progressive lifting of lockdown from 6:00 a.m to 3:00 p.m	Atsinanana and Alaotra-Mangoro

* (Antananarivo).

** (Toamasina).

*** (Fianarantsoa).

**** (Moramanga, Ambatondrazaka, Anosibe an’Ala).

We illustrate that the current incidence of COVID-19 in Madagascar can be explained by the early and effective implementation of NPIs and low case detection rates, both of which are supported by strong anecdotal evidence. In contrast, arguments of regional-specific epidemiology are based on correlational observations that have yet to be proven. This suggests that the epidemic could grow in Madagascar, and similar countries in SSA, and that these populations remain at risk of an impending health crisis. Our model indicates that, if NPIs remain enforced at the level needed to explain current case burdens, nearly 50,000 lives could be saved. Even with NPIs, 30% of the Malagasy population could become infected by March 2021, making COVID-19 the leading killer in Madagascar over this epidemic period, hence the importance of conducting clinical trials and continually improving access to healthcare.

## Case detection

**Figure 1.**
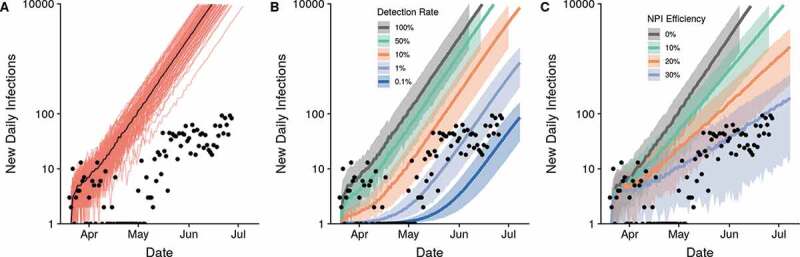
The lower-than-expected daily incidence can be explained by detection rates of 0.1–1% or NPI efficiencies of 30% alone. Predicted epidemic trajectories for the unmitigated scenario (a), range of detection rates (b), and range of NPI efficiencies (c). Results from 100 simulations are shown in A with the black line representing the median number of cases. Shaded regions represent the 95% confidence intervals around the median in panels B and C. All simulations began on the date of the first positive imported case in Madagascar, 20 March 2020. The y-axis is plotted on the log10-scale.

By July 2020, the simple forecast for an unmitigated epidemic predicts a daily incidence of 34,322 cases, which is nearly 500 times the reported daily incidence of 71.71 (7-day rolling average) (Figure 1(a)). Simply accounting for detection rates between 0.1% and 1% results in predictions that closely approximate the reported daily incidence of COVID-19 cases in Madagascar (Figure 1(b)). Are these low levels of case detection reasonable? For countries where per capita testing is over 100-fold higher than in Madagascar (79.1/100,000 population in July 2020), it is estimated that less than 10% of COVID-19 cases have been detected [29]. Though the precise case detection rates for Madagascar cannot be discerned from available data, there are a number of indicators suggesting that these are lower than the already low rates of Europe or the US.

First, case definitions in Madagascar may be stricter than elsewhere. In June 2020, about 12% of suspected cases tested were confirmed by RT-PCR in Madagascar compared to a positivity rate of less than 5% in Europe and the US over the same period [30,31]. In July 2020, this positivity rate was as high as 50% in Madagascar [32]. This suggests strict criteria for test eligibility (e.g. requiring symptoms and known positive contacts) is being used in Madagascar and many cases could potentially be missed. Further, strict diagnostic criteria may be a result of the original testing policy of Madagascar early in the epidemic, which required that suspected cases first test positive with antibody-based rapid detection tests (RDTs) before being confirmed through RT-PCR tests. The low probability of detection of antibodies early in the infectious period (i.e. the first week of symptoms) and the high rate of false-negatives with RT-PCR later in the infectious period (i.e. after 7–10 days post symptom onset) [33] create a short window for detection.

Second, the Madagascar health system itself is only receiving a portion of symptomatic COVID-19 cases. The proportion of asymptomatic COVID-19 cases is estimated to be between 40% and 45% [34]. In Madagascar, surveys indicate that 40.2% of people with respiratory infection symptoms seek healthcare [35], implying that over 50% of symptomatic COVID-19 infections may not even enter a public health facility. For COVID-like symptoms, this rate could be much lower given the stigma associated with the disease [11]. The combination of the high proportion of asymptomatic cases and low health-seeking behaviors suggest that, even if health centers test 50% of symptomatic COVID-19 cases attending a health facility, this would detect less than 25% of symptomatic cases.

Finally, there is limited diagnostic testing capacity in Madagascar, with RT-PCR testing available in five laboratories across three major cities. It is unlikely that health facilities in rural areas of the country, where nearly 50% of the population lives, are testing such a high percentage of cases. We can account for all of these factors to estimate an upper bound of detection rates for Madagascar (strict case definitions (0.3) x low proportion symptomatic (0.45) x low healthcare-seeking behaviors (0.2) x limited testing infrastructure (0.25)) to reasonably explain a detection rate in Madagascar of 1% or lower.

## Reduced transmission

A reduction in transmission rates of 30%, relative to an unmitigated scenario, can also explain the daily case report rates of COVID-19 in Madagascar (Figure 1(c)). This reduction could be the result of NPI policies put in place in Madagascar or of innate characteristics affecting the epidemiology of COVID-19 (e.g. baseline contact patterns, climate, etc.). NPIs were implemented within three days after the first confirmed imported case of COVID-19 in the country (Table 1), the majority of which focused on restricting intercity travel on roadways and included lockdowns in population centers. In contrast, the UK instituted a partial-lockdown on 23 March 2020, 52 days after the first confirmed case in the UK on 31 January 2020. The road system of Madagascar is highly fragmented, with most travel on a limited number of paved national roads that run North-South through the capital. Restricting travel on these roads has the potential to be highly effective in reducing human mobility in Madagascar, and therefore the spread of COVID-19. Further, most travel involves major population centers, particularly the capital city, Antananarivo [36]. These cities had much more stringent NPIs put in place early in the epidemic, including city-wide lockdowns and curfews (Table 1), and the targeted lockdown of these population centers could have reduced spread to the rest of the country. While mobility data is not available for Madagascar, other SSA countries have reported reductions in mobility ranging from 1.4% in Zambia to 19% in Senegal compared to pre-NPI levels [37]. In addition, Madagascar enforced the universal use of face masks in the first month following confirmation of the first cases, an intervention that is gaining increasing international consensus in the fight against COVID-19 [38]. With a sparse road network that is well regulated in Madagascar, in combination with city-wide temporary lockdowns and universal use of masks, 30% represents an obtainable reduction in contact rates.

Because NPIs were implemented early in the epidemic, their effects on transmission cannot be disentangled from baseline contact patterns in the country, which may be lower than those of Europe or the US. Nearly half (47.73%) of the Malagasy population lives in rural areas, and most of the country is over 3 hours from a population center with more than 50,000 people [39]. Therefore, baseline contact patterns in the rural areas of Madagascar may be reducing disease spread in a way that is unidentifiable from the effects of NPIs.

## Which path is Madagascar on?

**Figure 2.**
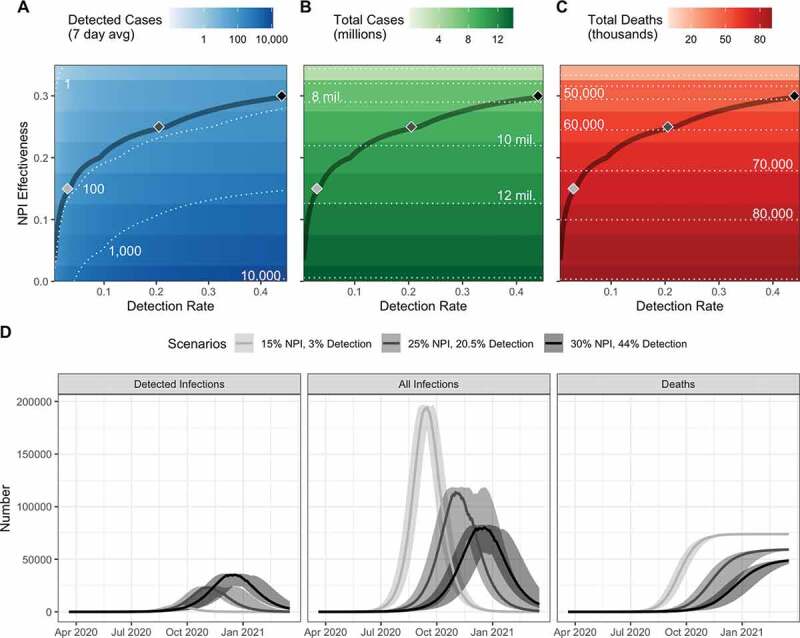
Low reported cases can be explained by different combinations of NPI effectiveness and detection rates. (a) The predicted number of daily cases (7 day average) that would be detected based on models of the epidemic at different combinations of NPI effectiveness and case detections rates. The dark contour line corresponds to the parameter space where the median number of predicted cases from 25 simulations equals the daily reported cases (7 day average) on June 22 (71.71 cases). High NPI effectiveness would thus require relatively high detection rates to explain the data based on these standard models. Similarly, if NPI were not effective, then the data could be explained with low detection rates. (b) Total cases after 1 year (approximating the final epidemic size) and (c) total deaths that correspond to the combination of NPI effectiveness and detection rates that explain daily cases in A. Shaded diamonds correspond to specific scenarios explored in panel D, illustrating the dynamics of detected infections, all infections, and cumulative deaths over the first year of the epidemic.

The evidence presented here provides no indication that the epidemiology (e.g. *R*_0_) of COVID-19 is fundamentally different in a fairly typical SSA country than elsewhere. We demonstrate that the current trend in reported cases in Madagascar can be explained by its early stage in the epidemic, combined with low detection rates and lower transmission rates from NPIs (Figure 2(a)). Understanding how much of the discrepancy between predicted and reported case burdens is due to low detection rates or NPIs has enormous implications for our expectations regarding the ‘true’ burden of COVID-19 in Madagascar. For this, we explored different combinations of detection rates and NPI efficacy that explain the observed trend in reported cases, together with associated predictions of epidemic morbidity and mortality burdens (Figure 2). If the low number of reported cases is due primarily to a low detection rate, we predict over 13 million people could be infected with the virus if NPIs are not in place (Figure 2(c,d)), imposing a huge burden on an already weakened health system. On the other hand, if the low number of cases is due to a reduction in contact patterns, the model predicts a lower total burden of approximately 8 million people infected with the virus (Figure 2(c,d)). If NPIs are driving these contact patterns and are responsible for the lower-than-expected case burden, the lifting of these restrictions is very likely to lead to an uncontrolled outbreak.

**Figure 3.**
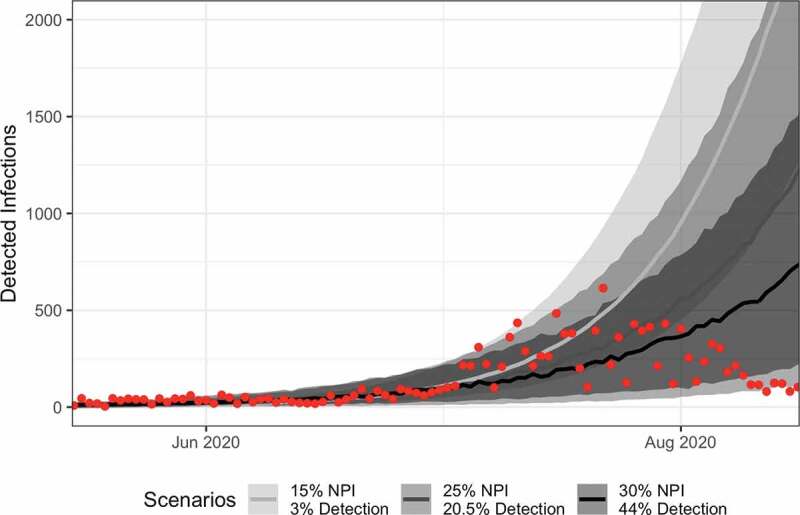
The simple modeled scenarios can accurately explain early, but not later, epidemic dynamics in Madagascar. Time series of predictions from the three scenarios explored in Figure 2D are plotted here (median and 95% CI), with line-shade corresponding to the scenario. Reported case data from the Madagascar Ministry of Health are plotted in the red points.

The initial modeling exercise was finalized on July 3 2020, using case data from March 20 2020 through June 27 2020. Following the initial publication review period, we re-evaluated the three scenarios considered in Figure 2(d) to understand whether these hypothesized explanations for low case numbers continue to explain epidemic dynamics in August 2020. While the current 7-day incidence rates do fall within the prediction bounds of the three scenarios, the epidemic curves do not align with reported case data (Figure 3). The current wave of infections has a decreasing trend, having peaked in late July, while the earliest peak in our scenarios was in October 2020. This suggests that the NPIs may have been more effective than our initial exploration exercise suggested, as our model framework required an NPI efficiency of 60% to suppress, rather than delay, the epidemic. Alternatively, it could reflect a transient phase in the epidemic. By mid-August, more than 75% of cases were reported in the densely populated Analamanga Region, which includes the capital city of Antananarivo. Countries with high heterogeneity in connectivity, such as Madagascar, may experience earlier-than expected peaks in the initial wave of the epidemic, with longer tails as asynchronous outbreaks occur in less-connected regions of the country [40]. This heterogeneity in connectivity may have similarly increased NPI effectiveness above the levels explored in our initial model, and will likely play a large role in determining epidemic dynamics as COVID-19 spreads into more rural areas of the country. Our model served as a tool to support the feasibility of proposed hypotheses in their explanation of the lower-than-expected case rates in Madagascar at the beginning of the epidemic, and its parameters are not fit to data from Madagascar. Therefore, it is outside the scope of our model to predict the immediate trajectory of the epidemic in Madagascar. Further modeling efforts are needed to incorporate human movement and regional heterogeneities in key parameters (e.g. contact and testing rates) to provide a more accurate picture of the epidemic. In addition, representative seroprevalence surveys can help fit model trajectories at particular points in time to better understand the evolution of the epidemic.

Although the global epidemic began several months ago, current infectious disease models for Madagascar and other SSA countries rely on limited data, resulting in disparate predictions. Pearson et al. [25] predicted a similar epidemic size for Madagascar as our model in an unmitigated scenario, with 75% of the population and nearly 100,000 deaths. In contrast, an analysis led by the WHO [12] predicted a total case burden nearly one third of this size (26% of the population) and only a fraction of COVID-19-related deaths (1,500). This study assumed that the regional particularities of SSA will decrease disease transmission and fatality rates based on country-specific proxies for these factors, such as climate, transportation networks, and contact matrices. Importantly, this study only considered *reductions* in transmission via reduced risks of exposure, with a maximum of 2.6% of the population of Madagascar at risk of exposure at any one time. While socio-ecological context is necessary to understand disease transmission, our exercise suggests that the difference between reported and predicted case burdens in SSA can be just as easily explained by accounting for low detection rates and NPIs that reduce interpersonal contact.

## Conclusion

We do not currently have enough evidence to suggest that the epidemiology of COVID-19 is different in Madagascar than elsewhere. The low number of reported cases can be explained by low detection rates, late introduction, and early and effective implementation of NPIs. In contrast to the theory of a salutary epidemiology, each of these explanations is supported by strong anecdotal evidence (Table 2). As lockdowns are gradually lifted, other NPIs, such as handwashing, social distancing, and face mask wearing, should be implemented to avoid a rapid growth in cases. The public health system should remain prepared for an outbreak, with a peak of infections expected between August and December depending on the transmission scenario (Figure 2(d)). The COVID-19 epidemic could become the leading public health problem in Madagascar, causing nearly twice as many deaths as are attributable to the current leading cause of death due to infectious disease, diarrheal disease [41]. It is important, therefore, to conduct clinical trials and continually improve access to health care. If NPIs remain in place at levels seen during the first months of the epidemic, the model suggests that this could prevent over 50,000 COVID-19 related deaths in Madagascar.

**Table 2.** Summary of evidence supporting or opposing three possible explanations for the low number of reported cases of COVID-19 in Madagascar.

	Supporting	Opposing
Low detection rates	High proportion of asymptomatic cases [34] Strict testing criteria Low healthcare seeking rates for acute respiratory infections [35] Diagnostic practices that limit the window of detection	Recent evaluation of health system preparedness via the International Health Regulations meant health systems were on high alert for an outbreak [20]
Epidemiological differences	Trained-immunity due to vaccinations or high prevalence of endemic disease could increase population’s resistance to infection [14] Transmission rate may be lower in sparsely populated areas [13] Virus survival is lower in humid, warm environments	Limited role for climate during pandemic phase of the outbreak [16] Past influenza outbreaks were not limited by sparse transport networks in SSA [17]
Early and effective NPIs	Lockdown in population centers implemented three days after first imported case Limiting travel on fragmented paved road network can easily disrupt within-country movement	

## Paper context

Countries in Sub-Saharan Africa have reported lower incidence and fatalities of COVID-19 than expected. There are several explanations for this: low case detection, differences in COVID-19 epidemiology, and early and effective non-pharmaceutical interventions (NPIs). We show that a combination of detection rates and NPIs explain the current incidence in Madagascar. There is little evidence for COVID-19 epidemiology to be different in SSA than elsewhere, and health systems remain at risk of an impending crisis.

## Appendix A. Modeling Methods

Reconciling model predictions with low reported cases of COVID-19 in Sub-Saharan Africa: Insights from Madagascar. 2020. Michelle V. Evans^1^, Andres Garchitorena^2,3^, Rado J.L. Rakotonanahary^3^, John M. Drake^1^, Benjamin Andriamihaja^3,4^, Elinambinina Rajaonarifara^2,3,5^, Calistus N. Ngonghala^6^, Benjamin Roche^2,7,8^, Matthew H. Bonds^3,9,^ Julio Rakotonirina^10^

We adapted an age-structured, stochastic, SEIR model (Roche et al. 2020) for the population of Madagascar. The population is divided into eight age classes *i*, within which they fall into the classic SEIR epidemiological compartments (Fig. S1). Individuals begin in the susceptible state (S), and are infected according to a force of infection that incorporates the transmission rate (*β*), contact rate between age classes (*θ*_ij_), and the number of infectious individuals in each age class (*I_j_* and *A_j_)*. Following an incubation period (1/ε), exposed individuals can then either become infected and asymptomatic with probability *p* or infected and symptomatic with probability *1-p*. Asymptomatic individuals then recover with a recovery rate (*σ*). We assume that asymptomatic individuals cannot become severe or die, but they do contribute to transmission equal to symptomatic individuals. Symptomatic-infected individuals recover at the same rate as asymptomatic individuals (*σ*) after which they enter a severe state (*S*) with probability π_i_, die with probability *α*_i_, or die from the virus (D_i_) with probability 1-π_i_-α_i_. Probabilities of case severity and mortality are age-dependent following Flaxman et al. 2020. There are no transitions between age classes and the primary purpose of the age classes is to account for age-structured contact rates and age-dependent severity and mortality rates. The model incorporates the age structure of Madagascar (Fig. S2A) (UNICEF 2018) and the contact matrix of Mozambique (Fig. S2B) (Prem et al. 2017), as no published contact matrix exists for Madagascar.

We calculate R_0_ following Roche et al. (2020):

Equation 1 $$R_{0}=\frac{\sum_{i}(\beta S_{i}\sum_{j}\theta_{ij})}{\varepsilon +\sigma }$$

The transmission rate (*β*) is set so that *R_0_* is 2.5, approximating values seen in Europe and the US (Kissler et al. 2020).

Simulations begin on 20 March 2020, with ten symptomatic infected individuals allocated to across age classes according to the age distribution of Madagascar’s population. The number of initial asymptomatic individuals in each age class is equal to the number of symptomatic cases divided by the proportion of symptomatic cases (1- *p*), resulting in 50 asymptomatic individuals allocated across age classes.

Each scenario was simulated 100 times for 365 days, with a timestep τ of 0.5 days. The unmitigated scenario used baseline parameters as described in Table 1, reporting the daily incidence of infected-symptomatic (I) and infected-asymptomatic (A) across all age classes as the total number of cases. Scenarios that explored lower detection rates scaled this number of total cases by the appropriate detection rate, applying an equal detection rate to symptomatic and asymptomatic cases and across all ages. Scenarios that explored reduced transmission via non-pharmaceutical interventions reduced the contact rates (*θ*_ij_) by multiplying this value by (1 – NPI efficiency). These reductions were applied equally across all age classes. Because the lockdown in Madagascar was announced on 23 March 2020, we began the contact reduction on day 4 of the simulations, corresponding to 24 March 2020. Our model assumes that these contact reductions remain constant for the duration of the epidemic (365 days).
